# The SOX4/EZH2/SLC7A11 signaling axis mediates ferroptosis in calcium oxalate crystal deposition-induced kidney injury

**DOI:** 10.1186/s12967-023-04793-1

**Published:** 2024-01-02

**Authors:** Xinzhou Yan, Yuqi Xia, Bojun Li, Zehua Ye, Lei Li, Tianhui Yuan, Baofeng Song, Weimin Yu, Ting Rao, Jinzhuo Ning, Fangyou Lin, Shuqin Mei, Zhiguo Mao, Xiangjun Zhou, Wei Li, Fan Cheng

**Affiliations:** 1 Department of Urology, Renmin Hospital, Wuhan University, Wuhan, 430060 Hubei People’s Republic of China; 2 Department of Nephrology, Shanghai Changzheng Hospital, Naval Medical University, Shanghai, 200003 People’s Republic of China

**Keywords:** Kidney stones, Kidney injury, Ferroptosis, EZH2, SOX4

## Abstract

Epigenetic regulation is reported to play a significant role in the pathogenesis of various kidney diseases, including renal cell carcinoma, acute kidney injury, renal fibrosis, diabetic nephropathy, and lupus nephritis. However, the role of epigenetic regulation in calcium oxalate (CaOx) crystal deposition-induced kidney injury remains unclear. Our study demonstrated that the upregulation of enhancer of zeste homolog 2 (EZH2)-mediated ferroptosis facilitates CaOx-induced kidney injury. CaOx crystal deposition promoted ferroptosis in vivo and in vitro. Usage of liproxstatin-1 (Lip-1), a ferroptosis inhibitor, mitigated CaOx-induced kidney damage. Single-nucleus RNA-sequencing, RNA-sequencing, immunohistochemical and western blotting analyses revealed that EZH2 was upregulated in kidney stone patients, kidney stone mice, and oxalate-stimulated HK-2 cells. Experiments involving in vivo EZH2 knockout, in vitro EZH2 knockdown, and in vivo GSK-126 (an EZH2 inhibitor) treatment confirmed the protective effects of EZH2 inhibition on kidney injury and ferroptosis. Mechanistically, the results of RNA-sequencing and chromatin immunoprecipitation assays demonstrated that EZH2 regulates ferroptosis by suppressing solute carrier family 7, member 11 (SLC7A11) expression through trimethylation of histone H3 lysine 27 (H3K27me3) modification. Additionally, SOX4 regulated ferroptosis by directly modulating EZH2 expression. Thus, this study demonstrated that SOX4 facilitates ferroptosis in CaOx-induced kidney injury through EZH2/H3K27me3-mediated suppression of SLC7A11.

**Graphical Abstract:**

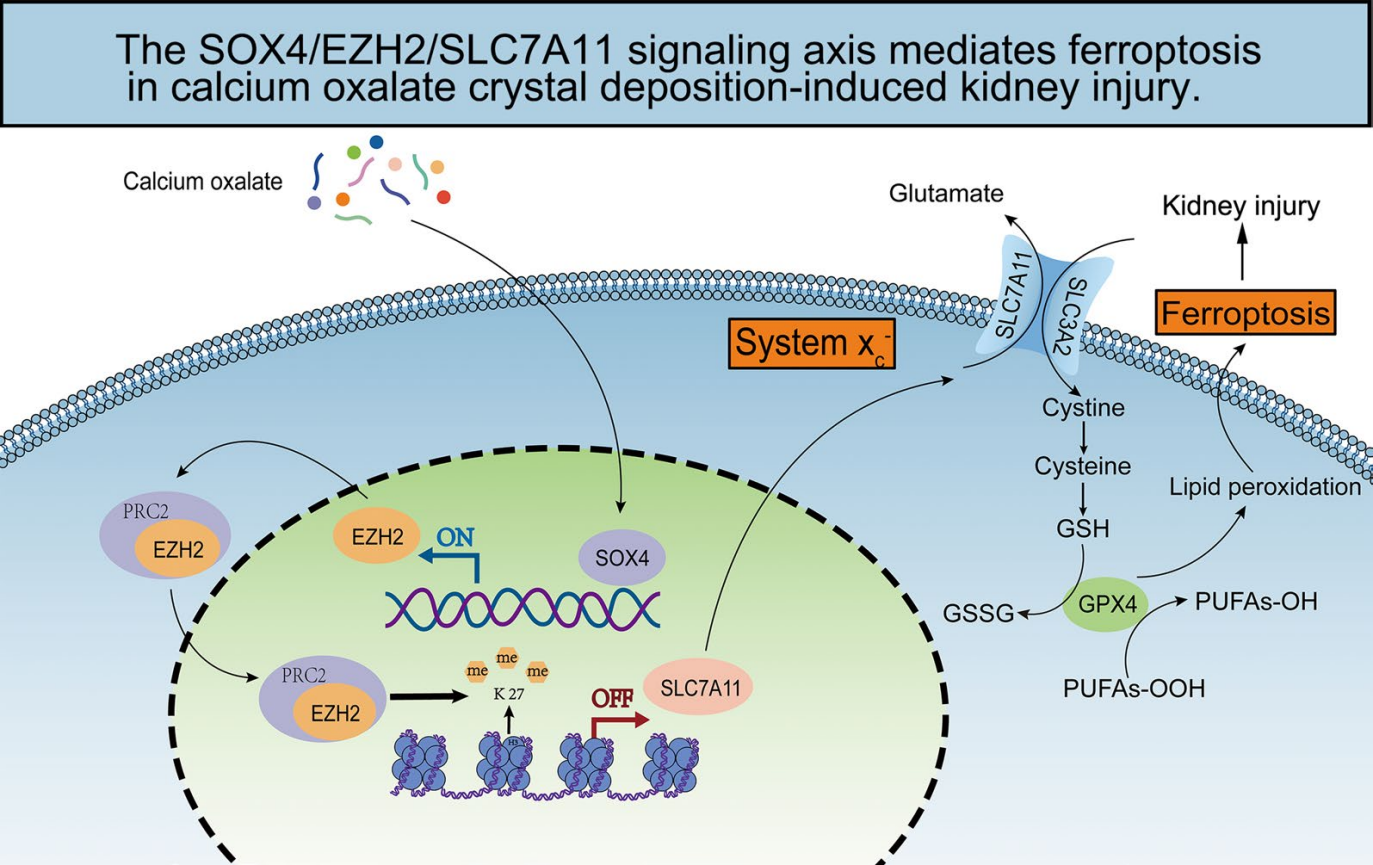

**Supplementary Information:**

The online version contains supplementary material available at 10.1186/s12967-023-04793-1.

## Introduction

Globally, kidney stones are linked to a high rate of incidence and recurrence, with incidence rates of 8.8% and 6.4% in the United States and China, respectively [1, 2]. The incidence of kidney stones has continued to increase [3]. Kidney stones can induce different degrees of kidney injury, including acute kidney injury (AKI) and chronic kidney disease (CKD), depending on the localization and dynamics of crystal deposition [4]. The most typical type of kidney stone is calcium oxalate (CaOx) stones. Patients with stones comprising ≥ 70% calcium content are classified as calcium stone formers [5]. Currently, the mechanism of CaOx-induced kidney injury is not fully elucidated. Previous studies have demonstrated that CaOx-induced renal tubular epithelial cell (TEC) apoptosis and necrosis may be the key mechanisms underlying the pathogenesis of kidney injury [6]. Therefore, exploring the relevant mechanisms of TEC injury-induced kidney injury is essential.

Moreover, CKD is highly associated with tubulointerstitial injury, which usually presents as renal interstitial fibrosis. Epithelial-mesenchymal transition (EMT) and endothelial–mesenchymal transition (EndMT) have been confirmed to play an important role in the process of renal fibrosis [7]. Particularly, EMT and EndMT have been confirmed to play a crucial role in diabetic renal fibrosis. Previous studies have shown that various signaling pathways, including TGF-β, Wnt, the Glucocorticoid receptor, Fibroblast Growth Factor Receptor 1 (FGFR1), and SIRT3, influence renal fibrosis by regulating EndMT. Furthermore, targeting EndMT has been confirmed to have a protective effect, and many clinical drugs, including SIRT3 and glycolysis inhibitors, empagliflozin, linagliptin, and N-acetyl-seryl-aspartyl-lysyl-proline (AcSDKP), all show anti-fibrotic therapeutic effects [8]. However, the effect of EMT and EndMT on CaOx-induced renal fibrosis remains unclear. Analogously, targeting EMT and EndMT may also play a protective role.

Ferroptosis, a unique kind of regulated cell death (RCD), is caused by the accumulation of iron-dependent lipid peroxide and reactive oxygen species (ROS) [9]. Glutathione (GSH) depletion and an increased Fe2+ transport rate lead to the accumulation of ROS. System Xc (−) comprises solute carrier family 7, member 11 (SLC7A11) and solute carrier family 3, member 2 (SLC3A2), regulating the synthesis of GSH. GSH activates glutathione peroxidase 4 (GPX4) to maintain the balance of the antioxidant system [10, 11]. In contrast to that induced by other RCDs, the cellular injury induced by ferroptosis is reversible [12]. According to various studies, ferroptosis is crucial to the etiology of many diseases, including AKI, CKD, and renal carcinoma [13, 14]. Recent studies have demonstrated that CaOx-induced ferroptosis activation promotes renal TEC and kidney injuries. Fe2 + levels and ROS levels increased while SLC7A11, GPX4, and GSH levels decreased in the oxalate-induced HK-2 cells [15]. Thus, inhibition of ferroptosis has a potentially protective effect in the treatment of kidney stones. However, the exact effect of ferroptosis on CaOx-induced kidney injury remains unclear.

Epigenetic modifications refer to the covalent modifications of DNA and histone proteins, which alter the phenotype without modifying the gene sequence [16]. The mechanisms underlying epigenetic regulation include the modification of histones, non-coding RNAs, and DNA methylation [17]. Types of histone modifications include methylation, acetylation, phosphorylation, and ubiquitination. Enhancer of zeste homolog 2 (EZH2), a part of polycomb repressive complex 2 (PRC2), promotes the trimethylation of histone H3 lysine 27 (H3K27me3), resulting in the suppression of downstream genes [18]. EZH2 inhibitors are reported to exert therapeutic effects on various kidney diseases, including renal cell carcinoma, AKI, renal fibrosis, diabetic nephropathy, lupus nephritis, and renal transplant rejection [19, 20]. The transcription factor SOX4, a member of the SOX family, has a significant impact on growth and development [21]. Various studies have confirmed the function of the SOX4/EZH2 axis in cancer models [22, 23]. This research sought to assess the possible impact of the SOX4/EZH2 axis on ferroptosis in the pathogenesis of CaOx-induced kidney injury and evaluate the protective effect of EZH2 inhibition.

## Materials and methods

### Clinical specimens

The kidney samples were collected from the Renmin Hospital of Wuhan University. Patients with CaOx kidney stones provided samples for the kidney stone group from their non-functioning kidneys; individuals who had a complete excision of their kidneys provided samples for the control group from their non-tumorous kidneys. The Renmin Hospital Ethics Committee at Wuhan University gave its approval to this project (WDRY2021-KS047). To take part in the study, each subject signed a formal waiver of informed consent. Each participant gave their written agreement to take part in the study.

### Animal experiments

**Fig. 1:**
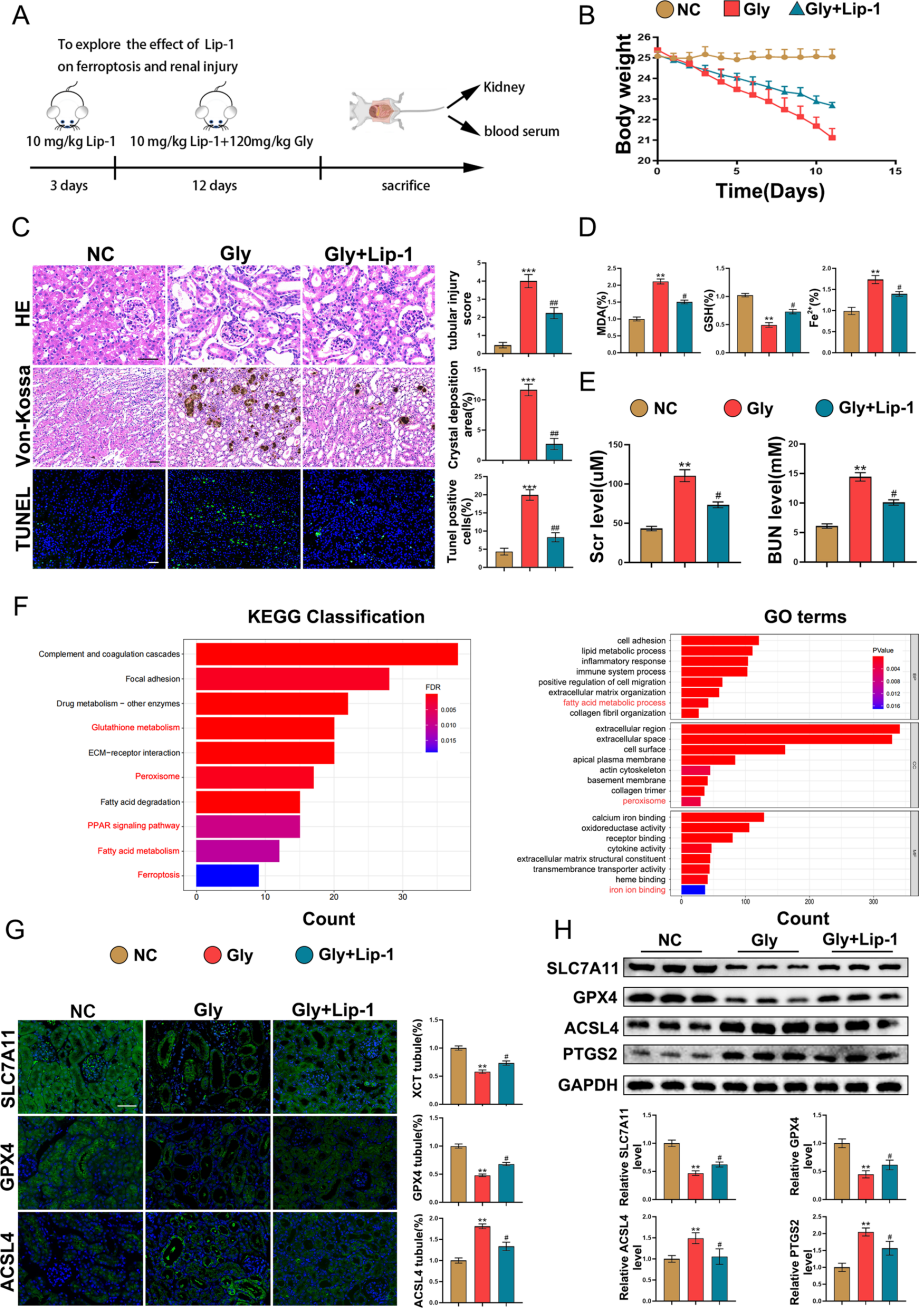
Ferroptosis is stimulated in kidney stone mice, and ferroptosis inhibition mitigates Gly-induced kidney injury. **A** Diagram of mouse moulding. **B** Effects of Lip-1 on bodyweight (n = 6). **C** H&E, Von-Kossa, and TUNEL staining (n = 6). **D** MDA, GSH, and Fe2 + levels (n = 6). **E** Serum BUN and Cr levels (n = 6). **F** GO and KEGG enrichment analyses of DEGs between the Gly and control groups (n = 3). **G** Immunofluorescence of SLC7A11, GPX4, and ACSL4 in vivo (n = 6). **H** Western blotting analysis of SLC7A11, GPX4, ACSL4 and PTGS2. Scale bar = 50 µm. *P < 0.05, **P < 0.01, and ***P < 0.001 compared with the control group; ^#^P < 0.05 and ^##^P < 0.01 compared with the Gly group

The Laboratory Animal Welfare and Ethics Committee authorized all animal research (WDRM-20200604). C57/BL6 wild-type mice aged 6–8 weeks (bodyweight = 23–26 g) were acquired from the Wuhan University Center for Animal Experiment. Mice were provided with sufficient food and water and kept in a suitable environment. Numerous studies have supported the crucial role of EZH2 in the early stages of embryonic development [24, 25]. Thus, to avoid the adverse effects of EZH2 inhibition, tamoxifen-inducible EZH2-knockout (iKO) mice were generated. EZH2^fl/fl^ mice, which were obtained from Prof. Xi Wang (Department of Immunology, School of Basic Medical Sciences; Advanced Innovation Center for Human Brain Protection, Beijing Key Laboratory for Cancer Invasion and Metastasis, Department of Oncology, Capital Medical University, Beijing, China), served as the control group. CAG-creER mice were from our previous studies [26, 27]. Finally, CAG-creER-EZH2^fl/fl^ (EZH2iKO) mice were constructed and injected with tamoxifen (75 mg/kg bodyweight) at week 6 to obtain EZH2 knockout mice. Mice were injected with glyoxylic acid (Gly; 120 mg/kg bodyweight; G10601; Sigma–Aldrich) for 12 days to create the kidney stone mice [28, 29]. The impact of ferroptosis inhibition on CaOx-induced kidney injury was examined by injecting mice with liproxstatin-1 (Lip-1; 10 mg/kg bodyweight; S7699; Selleck) for 15 days [30]. The experimental design is shown in Fig. 1. To further verify the effect of EZH2 inhibition on CaOx-induced kidney injury, GSK-126 (50 mg/kg bodyweight; HY-13470; MCE) were used every other day for 12 days [31].

### Cell culture

HK-2 cells, which were collected from Procell Life Science & Technology Co., Ltd., Wuhan, China, were cultivated in a sterile culture flask containing MEM accompanied by 10% fetal bovine serum (Gibco, Waltham, MA, United States) at 5% CO2 and 37 ℃. To explore the effect of oxalate concentration on EZH2 expression levels, cells were incubated with varying concentrations of oxalate for 24 h. The optimal concentration of oxalate was determined to be 2 mM. To investigate the effects of ferroptosis inhibition, cells were incubated with 0.5 µM Lip-1 and 2 mM oxalate for 24 h. The EZH2 knockdown lentiviral construct was obtained from Genepharma (Suzhou, China). The lentiviral sequences were as follows: LV2-negative control (NC), 5′-TTCTCCGAACGTGTCACGT-3′; LV2-EZH2, 5′-GCTCCTCTAACCATGTTTACA-3′. Virus-infected cells were exposed to 2 g/mL of puromycin in a solution. The clones were kept stable in a medium containing 1 g/mL puromycin for 72 h post-transfection. The short-interfering RNAs (siRNAs) were obtained from Sangon Biotech (Shanghai, China). The sequences of siRNAs were as follows: si-NC, 5′- UUCUCCGAACGUGUCACGUTT-3′ (sense) and 5′- ACGUGACACGUUCGGAGAATT-3′ (antisense); si-SLC7A11, 5′-CAUCCAGGAGUUAUGUUUATT-3′ (sense) and 5′-UAAACAUAACUCCUGGAUGTT-3′ (antisense); si-SOX4, 5′-AGCGACAAGAUCCCUUUCAUUTT-3′ (sense) and 5′-AAUGAAAGGGAUCU.

UGUCGCUTT-3′ (antisense). Lipofectamine 2000 (Invitrogen, USA) was used to transfect siRNAs into overnight-plated cells in a 6-well plate.

### qRT-PCR

Total RNA was extracted from tissues and cells using the TRIzol reagent (Invitrogen Life Technologies). Using the Takara reagent kit (Takara Biotechnology), the recovered RNA was then reverse-transcribed into complementary DNA. qRT-PCR analysis was performed with SYBR Green master mix (Yeasen). Additional file 1: Table S1 displays the qRT-PCR analysis primers used.

### Western blotting

Renal tissues and HK-2 cells were lysed using radioimmunoprecipitation assay buffer (Servicebio). The lysates were subjected to polyacrylamide gel electrophoresis and transferred to PVDF membranes. The membrane was occluded with 5% skim milk to prevent nonspecific binding, followed by overnight incubation with primary antibodies. Next, the membrane was washed with TBST and incubated with HRP-conjugated secondary antibodies for 1 h. Immunoreactive signals were formulated using an enhanced chemiluminescence kit (Biosharp, China) and visualized using a ChemiDoc MP system. We used ImageJ to calculate the relative protein density. Every experiment was conducted with three replicates. The following are the primary antibodies used in the analysis: anti-EZH2 (5246S; 98 kDa; 1:1000; CST), anti-H3K27me3 (9733S; 17 kDa; 1:1000; CST), anti-H3 (17,168–1-AP; 17 kDa; 1:2000; Proteintech), anti-SOX4 (A10717; 47 kDa; 1:1000; Abclonal), anti-SLC7A11 (A2413; 55 kDa; 1:1000; Abclonal), anti-GPX4 (A11243; 19 kDa; 1:1000; Abclonal), anti-acyl-CoA synthetase long-chain family 4 (ACSL4) (A6826; 79 kDa; 1:1000; Abclonal), anti-prostaglandin-endoperoxide synthase 2 (PTGS2) (66,351–1-lg; 68 kDa; 1:1000; Proteintech), and anti-GAPDH (10,494–1-AP; 37 kDa; 1:5000; Proteintech) antibodies.

### Renal function evaluation

To obtain serum, blood samples from mice were centrifuged at 3000*g* for 20 min. The serum levels of creatinine (Cr) and blood urea nitrogen (BUN), which served as markers for renal function, were analyzed using the appropriate kit (JianCheng, Nanjing, China).

### Histological analysis

The kidney tissues were embedded in paraffin and fixed with 4% paraformaldehyde. Renal tubular damage was analyzed using H&E staining as described previously [23]. Von Kossal staining was performed to detect CaOx crystal deposition. Using ImageJ, the crystal deposition area was measured.

### TUNEL assay

Tissue sections were prepared as described above. Proteinase K was employed to enhance the binding of the kit probe to damaged DNA in tissue sections. The samples were incubated with the TUNEL staining kit (C1086, Beyotime) at 37 °C for 1 h. Antifade mounting medium containing DAPI was used to encapsulate the samples. The TUNEL-positive rate was examined using an automated fluorescent microscope (magnification = 200×).

### Immunohistochemistry (IHC)

The tissue sections were dewaxed, rehydrated, and treated with 3% H2O2 for 10 min. The sections were then blocked with 5% bovine serum albumin (BSA) for 30 min and incub TUNEL ated at 4 °C for 12 h with anti-EZH2 antibodies. The sections were then incubated for 1 h at 37 °C with HRP-conjugated goat secondary antibodies. Immunoreactive signals were developed using 3,3′-diaminobenzidine. The positive signals in renal tissues were measured under 400× magnification. Ten consecutive, non-overlapping fields were analyzed blindly for each animal.

### Immunofluorescence

The sections were occluded for 30 min with 5% BSA and incubated with anti-SLC7A11, anti-GPX4, anti-ACSL4, anti-CD31, anti-Ecadherin, and anti-αSMA primary antibodies for 2 h, followed by incubation at 26 °C with specific secondary antibodies. DAPI was used to color the nuclei.

HK-2 cells were fixed with 4% paraformaldehyde for 20 min, permeabilized with 0.5% Triton X-100 for 15 min, and blocked with 5% BSA for 1 h. The cells were then incubated with anti-EZH2, anti-SLC7A11, anti-GPX4, and anti-ACSL4 primary antibodies overnight at 4 °C, followed by incubation with secondary antibodies. The images were captured under a fluorescent microscope and quantified using ImageJ.

### Malondialdehyde (MDA) and GSH assays

The MDA levels were evaluated using the MDA assay kit (S1031S, Beyotime). The GSH levels were evaluated using the GSH assay kit (S0053, Beyotime).

### Iron assay

The relative Fe2 + concentrations in tissues and cells were evaluated using the iron assay kit (Ab83366, Abcam).

### Transcriptome RNA sequencing

Total RNA was extracted from matched tissues of the control (n = 3) and Gly (n = 3) groups. BGI Group (Shenzhen, China) was responsible for RNA sequencing and data analysis. The differentially expressed genes (DEGs) were screened using the R studio (DESeq2, lg|FC|> 1, FDR < 0.05). Furthermore, a volcano map was constructed with DEGs, and DEGs were subjected to Gene Ontology (GO) and Kyoto Encyclopedia of Genes and Genomes (KEGG) analyses. The single-nucleus RNA-seq dataset was obtained from the Kidney Precision Medicine Project (https://www.kpmp.org/) to compare the EZH2 expression level between patients with CKD and healthy groups. GSE73680 was obtained to examine the correlation between SOX4 and EZH2 expression levels. In terms of cells, RNA sequencing and data analysis of the Ox + sh-NC (n = 3) and Ox + sh-EZH2 (n = 3) groups were conducted by the BGI Group. Ferroptosis-related upregulated DEGs were used to generate heatmaps.

### Transmission electron microscopy (TEM)

Fresh renal cortex tissues with a volume of 1 mm^3^ were rapidly preserved at 4 °C in an electron microscopy fixative. Tissues were sectioned into ultrathin (60–80 nm) sections and double-stained with uranium and lead. The RECs and the mitochondria were examined using TEM.

### Lipid peroxidation assessment

The relative cellular levels of ROS were measured using the fluorescent C11 BODIPY 581/591 lipid peroxidation probe (Maokangbio, MX5211-1 MG). Cells were incubated with 5 µM of the C11-BODIPY probe at 37 °C for 30 min. Images were captured and examined under a fluorescent microscope. The oxidized dyes were measured at 488 and 510 nm, respectively (fluorescein isothiocyanate filter). The reduced dyes were measured at 581 and 591 nm, respectively (Texas Red filter).

Using flow cytometry, lipid ROS levels were measured. The cell culture medium was replaced with 1 mL of medium containing 5 µL of C11-BODIPY dye, and the cells were incubated for 60 min and then rinsed twice with PBS and suspended in 500 µL PBS. The cell suspension was filtered through a cell strainer (70 nm) and subjected to flow cytometry to examine ROS levels.

### Chromatin immunoprecipitation (CHIP)-PCR and CHIP-qRT-PCR

Cells were incubated with 1% formaldehyde for 10 min at room temperature for cross-linking. Next, the cells were lysed using a lysis buffer and sonicated for 30 min. For immunoprecipitation, the supernatant was incubated with 5 g of anti-H3K27me3 and anti-EZH2 antibodies in Fig. 5 or anti-SOX4 antibody in Fig. 6. Rabbit IgG served as the control. The immunoprecipitated DNAs were identified using CHIP-PCR or CHIP-qRT-PCR. The primers are shown in Additional file 1: Table S2.

### Statistical analyses

GraphPad Prism (version 8.0) was utilized for all statistical analyses. Data are expressed as the mean ± standard deviation. A one-way analysis of variance was used to compare the group means. At P < 0.05, differences were deemed significant.

## Results

### Lip-1 mitigates CaOx-induced ferroptosis in vivo and oxalate-induced ferroptosis in vitro

A kidney stone mouse model was established to investigate the function of CaOx-induced ferroptosis in kidney injury and the protective impact of Lip-1 on kidney injury. Figure 1A illustrates the experimental design. As displayed in Fig. 1B, the Gly group experienced significantly greater weight loss than the control group. Treatment with Lip-1 mitigated Gly-induced weight loss. Analysis of BUN and Cr levels revealed that Lip-1 markedly suppressed CaOx-induced renal function impairment (Fig. 1E). H&E, Von-Kossa, and TUNEL staining further confirmed that Lip-1 mitigated CaOx-induced tubular injury, crystal deposition, and renal cell death (Fig. 1C). Next, tissues from the control (n = 3) and Gly (n = 3) groups were subjected to RNA sequencing to determine if ferroptosis was activated in the Gly group. RNA sequencing analysis revealed that the numbers of upregulated and downregulated DEGs were 1346 and 610, respectively. Furthermore, GO enrichment analysis demonstrated that DEGs in the Gly group were significantly enriched in the fatty acid metabolic process, peroxisome, and iron ion binding, which are highly associated with ferroptosis. KEGG enrichment analysis showed that DEGs were enriched in glutathione metabolism, peroxisome, PPAR signaling pathway, fatty acid metabolism, and ferroptosis, which are highly associated with our study (Fig. 1F). Immunofluorescence and western blotting analyses confirmed that ferroptosis was activated in the Gly group and that Lip-1 suppressed the Gly-induced activation of ferroptosis (Fig. 1G–H). Additionally, analysis of the MDA, GSH, and Fe2 + levels confirmed the protective effects of Lip-1 (Fig. 1D). To further investigate the activation of ferroptosis in vitro and the protective impact of Lip-1, western blotting and immunofluorescence analyses were conducted to examine the SLC7A11, GPX4, and ACSL4 levels. The outcomes of in vitro experiments paralleled those of in vivo experiments (Additional file 2: Figs. S1B–D). Furthermore, immunofluorescence of CD31/αSMA and Ecadherin/αSMA showed that Lip-1 suppressed CaOx-induced EMT and EndMT (Additional file 1: Fig. S1A). These findings indicate that Lip-1 can alleviate CaOx crystal deposition-induced kidney injury.

### EZH2 is upregulated in kidney stone patients, kidney stone mice, and oxalate-stimulated HK-2 cells

**Fig. 2:**
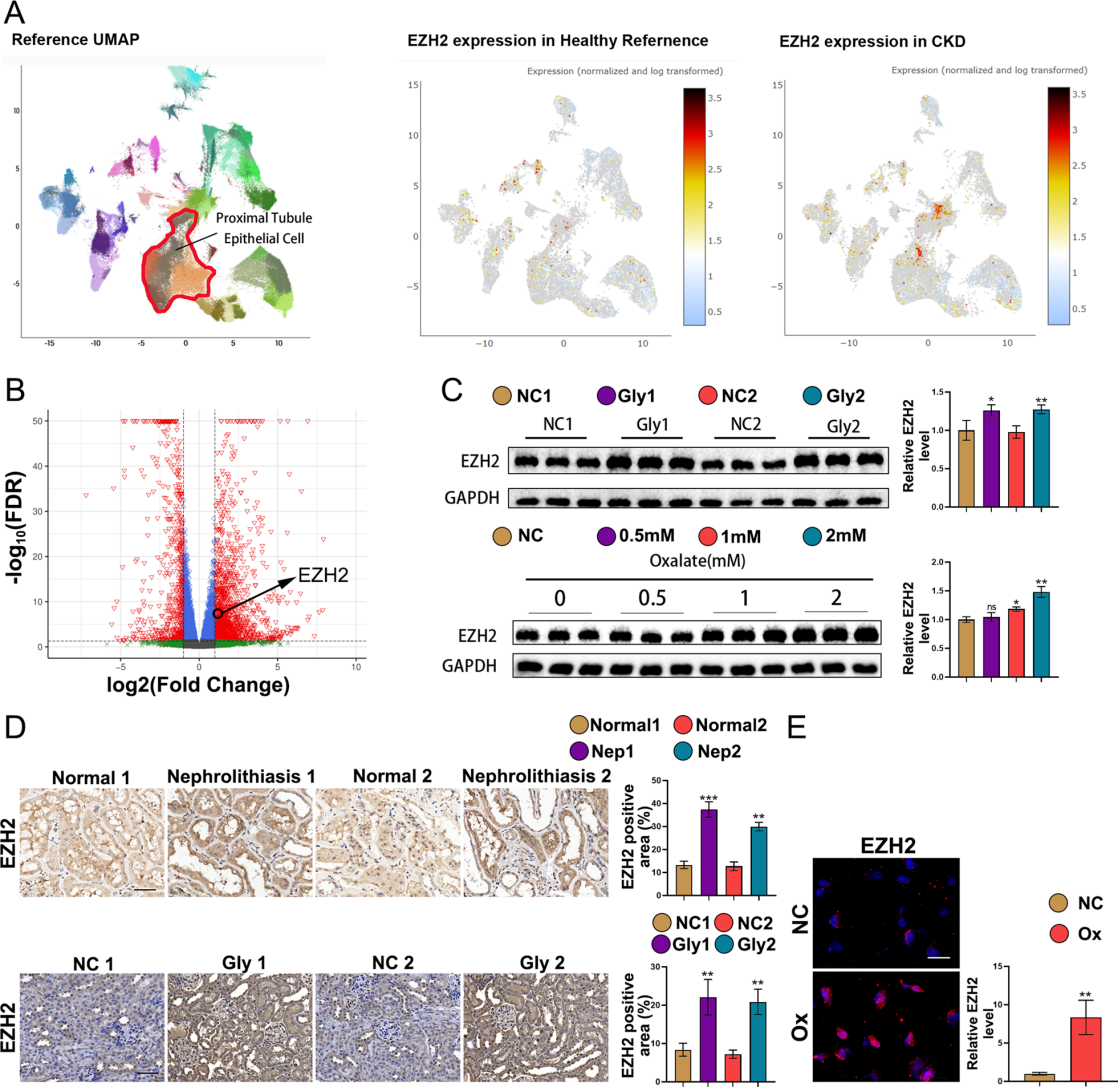
EZH2 is upregulated in kidney stone patients, kidney stone mice, and oxalate-stimulated HK-2 cells. **A** Single-nucleus RNA sequencing dataset analysis of CKD patients and healthy controls. **B** Volcano map of the DEGs between the Gly and control groups. **C** Western blotting quantitative analysis of EZH2 in vivo and in vitro. **D** Expression levels of EZH2 in kidney stone patients, kidney stone mice, and the control groups were examined using immunohistochemical analysis. **E** Immunofluorescence analysis of EZH2. Scale bar = 50 µm. *P < 0.05 and **P < 0.01 compared with the control group

Several studies have shown that EZH2 expression levels are upregulated in numerous animal models of kidney injuries, including AKI, renal fibrosis, lupus nephritis, and renal tumors [14]. Our study sought to explore the function of EZH2 in a kidney stone model. Analysis of the single-nucleus RNA sequencing dataset revealed that, compared to those observed in healthy controls, EZH2 expression levels were upregulated in patients with CKD, especially in proximal TEC (Fig. 2A). The volcano map of DEGs is shown in Fig. 2B. The upregulation of EZH2 in the Gly group was a characteristic feature. As shown in Fig. 2D, IHC analysis of human and mouse kidney tissues confirmed that EZH2 expression levels in kidney stone patients and kidney stone mice were higher than the controls. Consistently, immunofluorescence analysis showed that EZH2 was upregulated in oxalate-stimulated HK-2 cells (Fig. 2E). The upregulated EZH2 exhibited both cytoplasmic and nuclear expression. Furthermore, Fig. 2B shows the increased EZH2 expression in the Gly group mice. The outcomes of in vitro experiments were similar to those of in vivo experiments, although EZH2 was not significantly affected by low concentrations of oxalate treatment (Fig. 2G). Thus, the expression level of EZH2 is upregulated in kidney stone patients, kidney stone mice, and oxalate-stimulated HK-2 cells.

### EZH2 knockout alleviates CaOx-induced kidney injury and ferroptosis

**Fig. 3:**
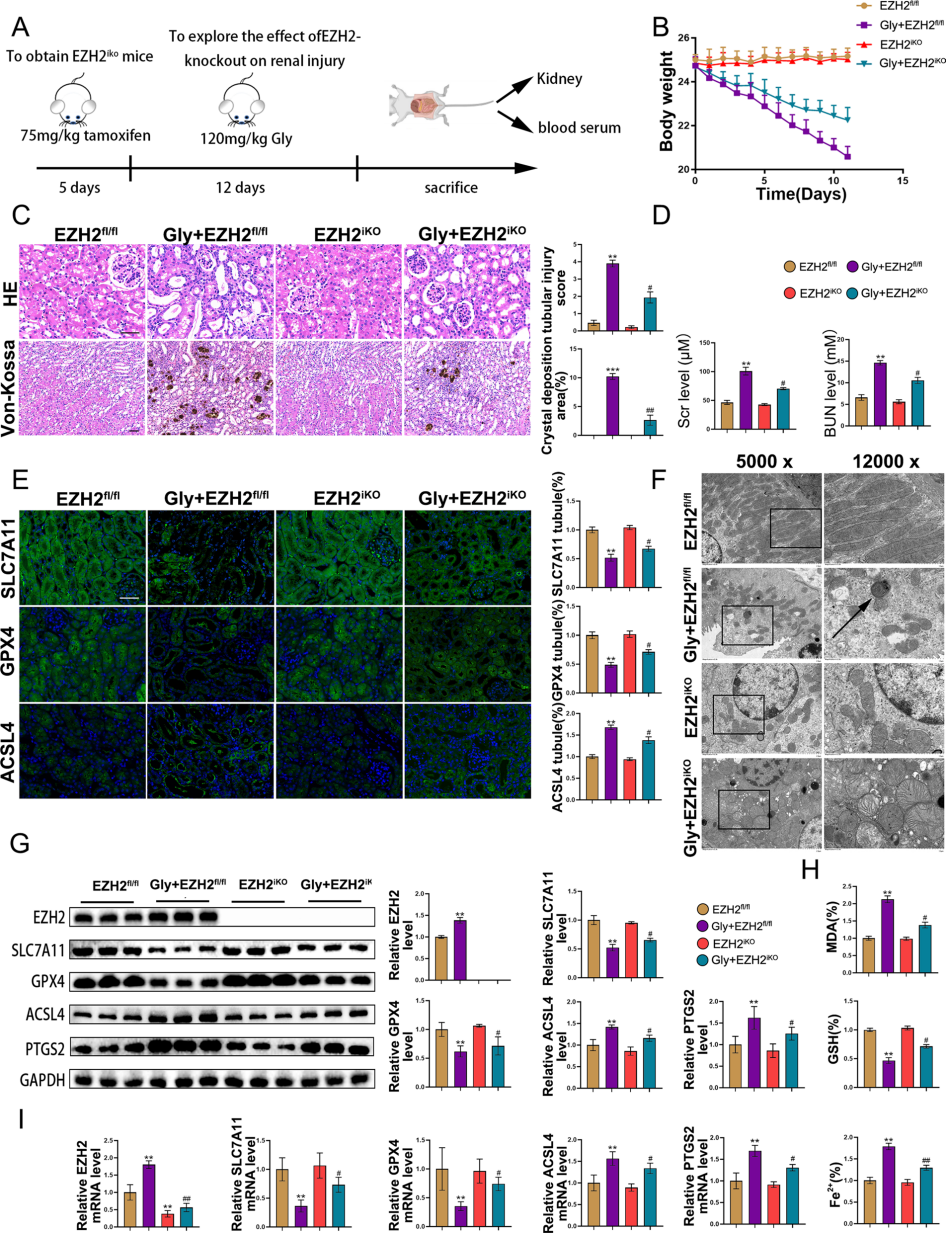
EZH2 knockout alleviates CaOx-induced kidney injury and ferroptosis. **A** Diagram of mouse moulding. **B** Effect of EZH2 knockout on bodyweight (n = 6). **C** H&E and Von-Kossa staining (n = 6). **D** Serum BUN and Cr levels in different groups (n = 6). **E** Immunofluorescence analysis of SLC7A11, GPX4, and ACSL4 in kidney tissues (n = 6). **F** Representative images were acquired using a TEM. **G** Western blotting analysis of EZH2, SLC7A11, GPX4, ACSL4 and PTGS2. **H** Relative MDA, GSH, and Fe2 + levels (n = 6). **I** EZH2, SLC7A11, GPX4, and ACSL4 mRNA levels were testing using qRT-PCR. Scale bar = 50 µm. *P < 0.05, **P < 0.01, and ***P < 0.001 compared with the EZH2^fl/fl^ group; ^#^P < 0.05, ^##^P < 0.01, and ^###^P < 0.01 compared with the Gly + EZH2^fl/fl^ group

Based on the activation of ferroptosis and the upregulation of EZH2 in kidney stones, we hypothesized that EZH2 may modulate CaOx-induced ferroptosis and consequently promote kidney injury. To validate the protective effect of EZH2 inhibition, tamoxifen-induced knockout of EZH2 (EZH2^iKO^) mice were established. EZH2^fl/fl^ mice served as the controls. Mice were first injected with tamoxifen (75 mg/kg bodyweight) for 5 days, and then Gly (120 mg/kg bodyweight) for 12 days (Fig. 3A). The weight loss was not significantly different between the EZH2^iKO^ and EZH2^fl/fl^ mice. However, the CaOx-induced weight loss in EZH2iKO mice was obviously less than that in EZH2^fl/fl^ mice (Fig. 3B). As shown in Fig. 3D, the CaOx-induced BUN and Cr levels in EZH2iKO mice were markedly downregulated when compared with those in EZH2^fl/fl^ mice. H&E and Von-Kossa staining showed that tubular damage and crystal deposition were more prevalent in the Gly group. The knockout of EZH2 significantly decreased Gly-induced tubular injury and crystal deposition (Fig. 3C). To further explore the impact of EZH2 knockout on ferroptosis, mitochondrial morphology was examined using TEM. We found that the mitochondria exhibited a swollen and shrunken morphology with increased membrane density in the Gly group. EZH2 knockout markedly suppressed these changes (Fig. 3F). Immunofluorescence and western blotting analyses of SLC7A11, GPX4, ACSL4, and PTGS2 indicated that the knockout of EZH2 inhibited CaOx crystal deposition-induced ferroptosis (Figs. 3E, G). The qRT-PCR results were consistent with western blotting analysis (Fig. 3I). Additionally, analysis of MDA, GSH, and Fe2 + levels confirmed the inhibitory effect of EZH2 knockout on ferroptosis (Fig. 3H). Furthermore, immunofluorescence of CD31/αSMA and Ecadherin/αSMA showed that knockout of EZH2 suppressed CaOx-induced EMT and EndMT (Additional file 3: Fig. S2A). These findings demonstrated that EZH2 knockout suppressed CaOx-induced kidney injury and ferroptosis.

### EZH2 knockdown mitigates oxalate-induced ferroptosis in vitro

**Fig. 4:**
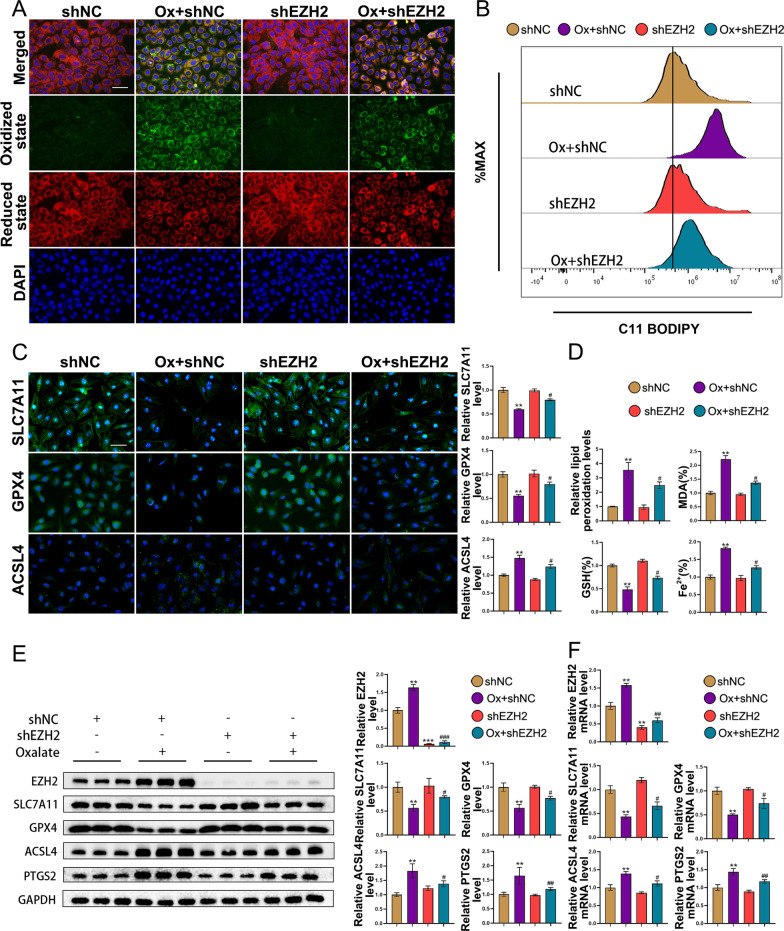
EZH2 knockdown mitigates oxalate-induced ferroptosis in HK-2 cells. **A** The effect of EZH2 knockdown on lipid peroxidation in vitro. **B** Effects of EZH2 knockdown on lipid ROS levels were analyzed using the C11-BODIPY fluorescence probe. **C** Immunofluorescence analysis of SLC7A11, GPX4, and ACSL4. **D** Relative lipid peroxidation, MDA, GSH, and Fe2 + levels in HK-2 cells. **E** Western blotting analysis of EZH2, SLC7A11, GPX4, ACSL4 and PTGS2. **F** The mRNA levels of EZH2, SLC7A11, GPX4, ACSL4 and PTGS2. Scale bar = 50 µm. *P < 0.05, **P < 0.01, and ***P < 0.001 compared with the shNC group; ^#^P < 0.05, ^##^P < 0.01, and ^###^P < 0.001 compared with the Ox + shNC group

A lentiviral shRNA construct of EZH2 was generated to validate the impact of EZH2 on ferroptosis in vitro. As shown in Fig. 4A, C11 BODIPY staining revealed that oxalate-treated HK-2 cells exhibited strong green fluorescence. The knockdown of EZH2 decreased the green fluorescence. The quantification of fluorescence intensity is shown in Fig. 4D. Lipid ROS generation is the main characteristic of ferroptosis. Flow cytometric analysis revealed that EZH2 knockdown mitigated the oxalate-induced upregulation of ROS (Fig. 4B). Immunofluorescence analysis of SLC7A11, GPX4, and ACSL4 revealed that oxalate activated ferroptosis, which was suppressed upon EZH2 knockdown (Fig. 4C). Analysis of MDA, GSH, and Fe2 + levels confirmed the effect of EZH2 knockdown on ferroptosis (Fig. 4D). These outcomes were similar to western blotting and qRT-PCR analyses (Figs. 4E, F). Thus, the impact of EZH2 knockdown in vitro was consistent with that of in vivo experiments.

### EZH2 regulates ferroptosis in HK-2 cells by directly suppressing SLC7A11 expression

**Fig. 5:**
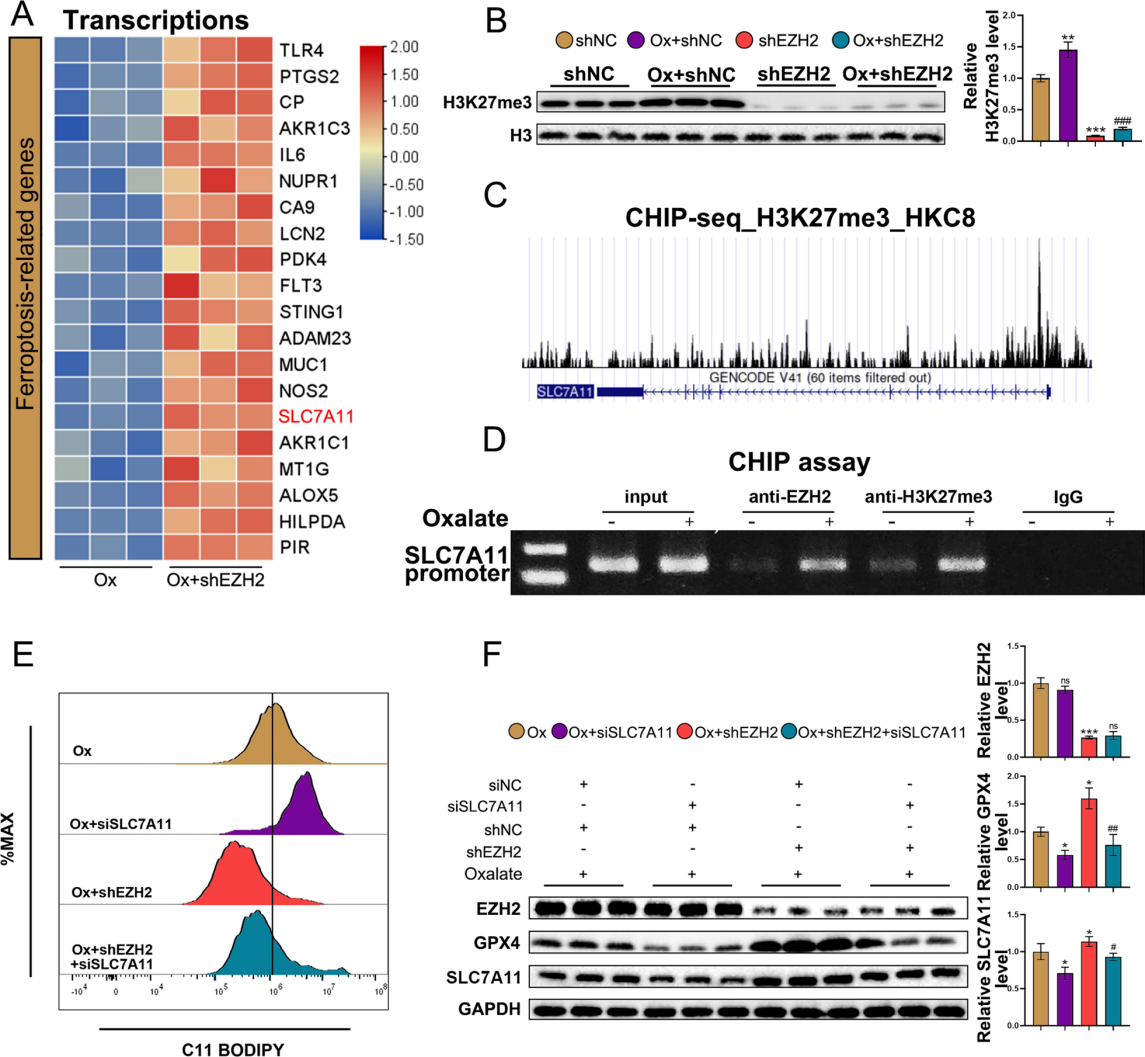
EZH2 regulates ferroptosis by directly suppressing SLC7A11 expression. **A** Heat map showed the genes significantly upregulated in the shEZH2 group. Red and blue colors denote ferroptosis-related upregulated and downregulated genes, respectively. **B** Western blotting analysis of H3K27me3. **C** Genome browser view of the H3K27me3 density at the SLC7A11 promoter region. **D** Analysis of EZH2-SLC7A11 promoter interaction using the CHIP assay. **E** SLC7A11 inhibition reverses the effects of the EZH2 knockdown on ferroptosis. The lipid ROS levels were analyzed using the C11-BODIPY probe. **F** Western blotting analysis of EZH2, SLC7A11, and GPX4. **P < 0.01, ***P < 0.001 compared with the shNC group; ^###^P < 0.001 compared with the Ox + shNC group in **B**. *P < 0.05 and **P < 0.01 compared with the Ox group, ^#^P < 0.05 and ^##^P < 0.01 compared with the Ox + shEZH2 group in F

EZH2 is reported to suppress gene expression by inducing trimethylation of H3K27. Western blotting analysis revealed that EZH2 knockdown mitigated the oxalate-induced upregulation of H3K27me3 (Fig. 5B). To screen genes directly affected by EZH2, matched oxalate-stimulated HK-2 cells (n = 3) and oxalate-stimulated EZH2 knockdown HK-2 cells (n = 3) were subjected to RNA sequencing. As shown in Fig. 5A, 20 genes, including SLC7A11, were significantly upregulated in the oxalate + sh-EZH2 group and were related to ferroptosis. Therefore, we hypothesized that EZH2 may affect ferroptosis by modulating SLC7A11 expression. Analysis of the online CHIP sequencing dataset revealed the enrichment of H3K27me3 at the SLC7A11 promoter (Fig. 5C). Furthermore, the CHIP assay revealed that the expression of SLC7A11 was regulated via EZH2-mediated epigenetic silencing in oxalate-stimulated HK-2 cells (Fig. 5D). To investigate the function of EZH2-mediated SLC7A11 suppression in CaOx crystal deposition-mediated ferroptosis, recovery experiments were performed with oxalate-stimulated HK-2 cells. SLC7A11 siRNA transfection promoted the oxalate-induced upregulation of ROS levels. Furthermore, the knockdown of SLC7A11 successfully rescued the sh-EZH2-mediated downregulation of ROS levels (Fig. 5E). Western blotting analysis showed that the knockdown of SLC7A11 did not influence the EZH2 protein level. In contrast, EZH2 knockdown significantly upregulated the SLC7A11 protein level, indicating that SLC7A11 functions downstream of EZH2. SLC7A11 siRNA rescued the sh-EZH2-induced upregulation of SLC7A11 and GPX4 protein levels (Fig. 5F). These findings suggest that EZH2 modulates ferroptosis by directly regulating the transcription of SLC7A11 in HK-2 cells.

### EZH2 is a direct transcriptional target of the transcription factor SOX4

**Fig. 6:**
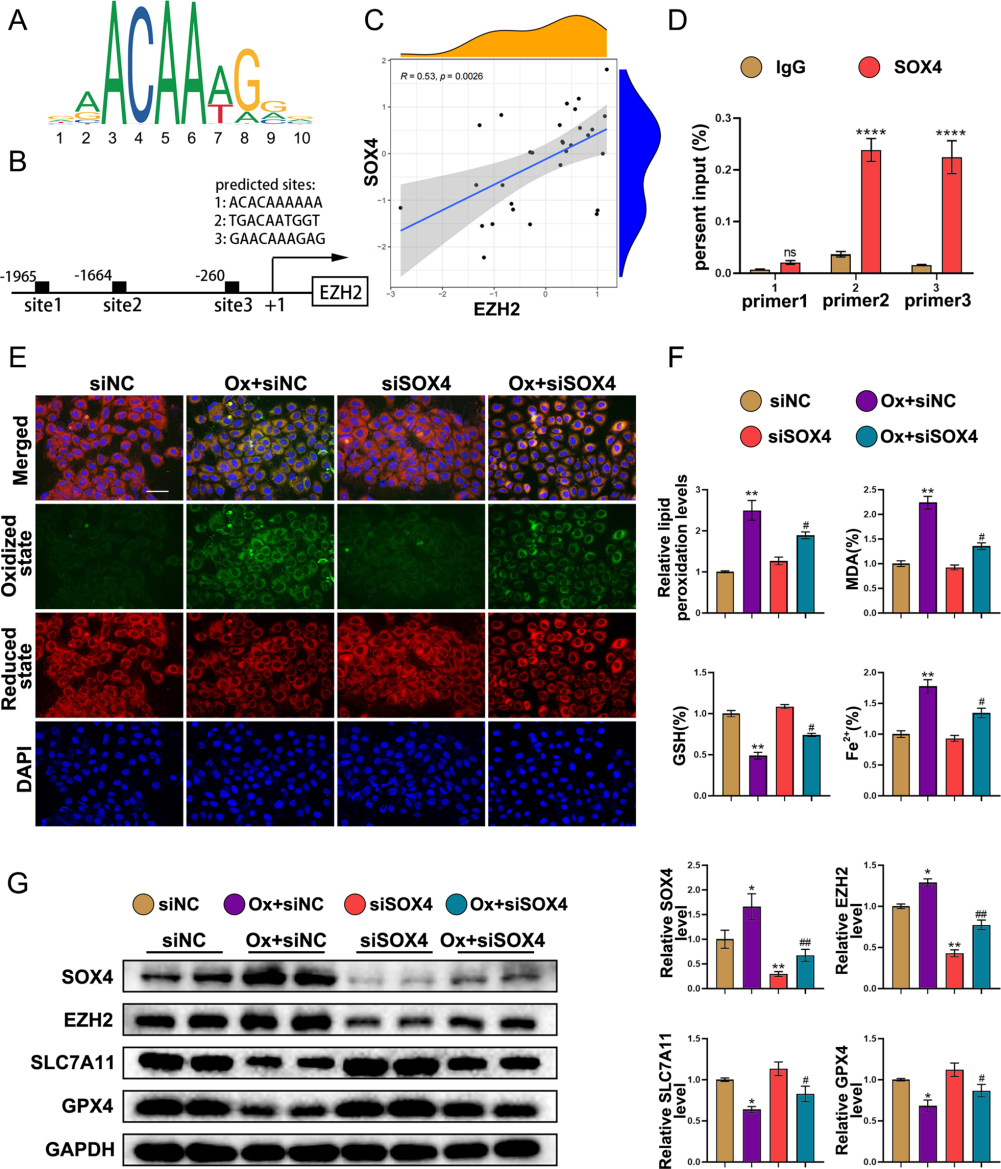
EZH2 is a direct transcriptional target of the transcription factor SOX4. **A** Sequence logo of SOX4. **B** Potential binding sites of SOX4 at the promoter of EZH2. **C** Correlation between SOX4 and EZH2 expression levels. The mRNA levels of SOX4 were correlated with EZH2 (r = 0.53; P = 0.0026). **D** Relative enrichment of SOX4 on the promoter region of EZH2 gene was evaluated by CHIP-qPCR in HK-2 cell. **E** The effect of SOX4 knockdown on lipid peroxidation. **F** Relative lipid peroxidation, MDA, GSH, and Fe2 + levels. **G** Western blotting analysis of SOX4, EZH2, SLC7A11, and GPX4. Scale bar = 50 µm. *P < 0.05 and **P < 0.01 compared with the siNC group; ^#^P < 0.05 and ^##^P < 0.01 compared with the Ox + siNC group

To explore the factors involved in upregulating EZH2 expression in kidney stones, the EZH2 promoter sequences were obtained from the NCBI database to predict the transcription factors to which EZH2 may bind. Analysis with the UCSC Genome Browser revealed 30 transcription factors, including SOX4 (Additional file 1: Table S3). Furthermore, RNA sequencing analysis of animal tissues revealed the upregulation of SOX4 in the Gly group. Hence, we hypothesized that SOX4 may regulate ferroptosis by modulating the transcription of EZH2 in oxalate-stimulated HK-2 cells. JASPAR software was utilized to forecast the binding site of SOX4 with a relative profile score threshold of > 80%. Figure 6A shows the sequence logo of SOX4, and Fig. 6B shows three putative SOX4-binding sites (− 1965 to − 1956 bp; − 1664 to − 1655 bp; − 260 to − 251 bp). To confirm the correlation between SOX4 and EZH2 expression levels, the GSE73680 dataset was downloaded. Correlation analysis was performed with the RNA sequencing data of 30 tissues, including 24 Randall’s plaques from kidney stone patients and 6 healthy papillary tissues from the control groups. EZH2 was positively correlated with SOX4 (r = 0.53; P = 0.0026) (Fig. 6C). Furthermore, CHIP-qPCR and dual-luciferase analyses revealed that binding site three was responsible for SOX4-mediated EZH2 promoter activity (Figs. 6D and Additional file 3: Fig. S2B). C11 BODIPY staining of oxalate-stimulated HK-2 cells revealed strong green fluorescence, indicating the upregulation of lipid peroxidation, while the knockdown of SOX4 reduced the intensity of green fluorescence (Fig. 6E). Analysis of MDA, GSH, and Fe2+ levels confirmed the suppressive effect of SOX4 knockdown on ferroptosis (Fig. 6F). Furthermore, western blotting analysis revealed that oxalate stimulation upregulated the SOX4 protein level. Additionally, the knockdown of SOX4, which is the upstream factor of the EZH2/SLC7A11 axis, downregulated the expression of EZH2 but upregulated SLC7A11 and GPX4 levels (Fig. 6G). These outcomes show that SOX4 regulates ferroptosis by modulating the expression of SLC7A11 through EZH2-mediated epigenetic suppression.

### GSK-126 suppresses Gly-induced ferroptosis

**Fig. 7:**
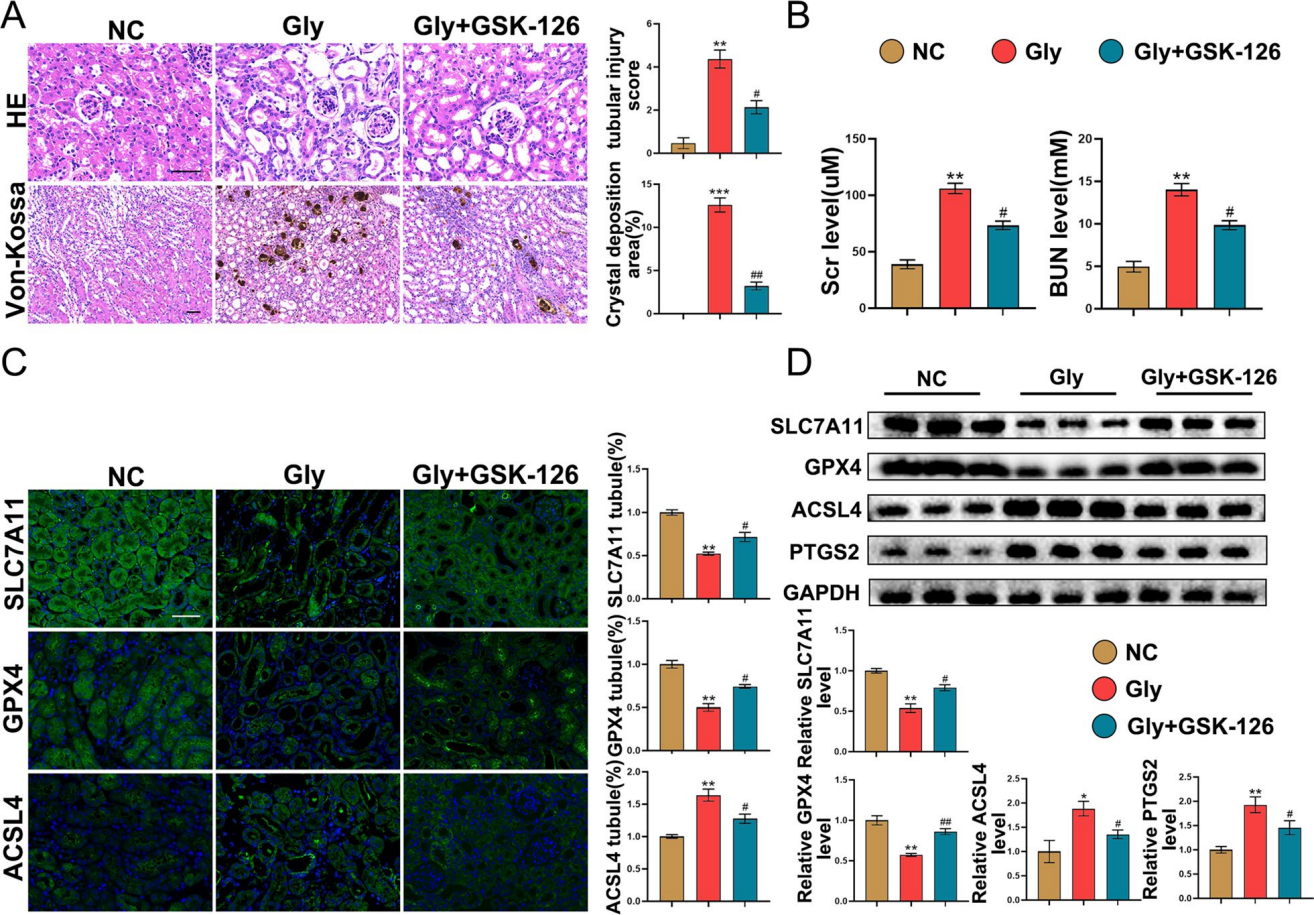
GSK-126 suppresses Gly-induced ferroptosis. **A** Tubular injury and CaOx crystal deposition were evaluated using H&E and Von-Kossa staining (n = 6). **B** Serum levels of BUN and Cr in different groups (n = 6). **C** Immunofluorescence analysis of SLC7A11, GPX4, and ACSL4 in kidney tissues (n = 6). **D** Western blotting analysis of SLC7A11, GPX4, ACSL4 and PTGS2 in kidney tissues. Scale bar = 50 μm. *P < 0.05, **P < 0.01, and ***P < 0.001 compared with the control group; ^#^P < 0.05 and ^##^P < 0.01 compared with the Gly group

GSK-126 is a classical EZH2 inhibitor. To further confirm the impact of EZH2 inhibition on ferroptosis, animals in the Gly group were injected with GSK-126. As shown in Fig. 7A, B, GSK-126 suppressed Gly-induced renal function impairment, tubular injury, and crystal deposition. Furthermore, immunofluorescence and western blotting analyses further confirmed that GSK-126 suppressed Gly-induced ferroptosis (Fig. 7C, D). Moreover, immunofluorescence of CD31/αSMA and Ecadherin/αSMA showed that GSK-126 significantly suppressed CaOx-induced EMT and EndMT (Additional file 3: Fig. S2C). These findings indicate that the EZH2 inhibitor GSK-126 can alleviate CaOx-induced ferroptosis.

## Discussion

EZH2 is reported to inhibit the transcription of downstream genes through epigenetic modifications. Recently, the protective effect of EZH2 inhibition has been demonstrated, and various EZH2 inhibitors have been approved for clinical cancer treatments [32]. For kidney injuries, EZH2 is also a potential therapeutic target. Previous studies have confirmed that EZH2 is upregulated in ischemia/reperfusion (I/R)-induced AKI [16, 33]. Moreover, the protective effect of EZH2 inhibition with 3-DZNep has been demonstrated in kidney injuries. In a model of sepsis-induced AKI, inhibition of EZH2 expression could reduce the apoptotic and inflammatory responses of renal TEC, alleviating AKI by mitigating the transcriptional inhibition of SOX9 [34]. Furthermore, EZH2 inhibition was proven to suppress cisplatin-induced renal TEC apoptosis and AKI by maintaining the expression level of E-calmodulin [35]. However, the exact function of EZH2 in CaOx-induced kidney injury has not been examined. This study demonstrated that EZH2 was significantly upregulated in kidney stone patients, kidney stone mice, and oxalate-stimulated HK-2 cells. EZH2 knockout in kidney stone mice and EZH2 knockdown in oxalate-stimulated HK-2 cells significantly mitigated the CaOx-induced kidney injury. Experiments with the EZH2 inhibitor GSK-126 also confirmed the protective effect of EZH2 inhibition. Therefore, EZH2 inhibition is likely to have a protective impact on CaOx-induced kidney injury.

Various studies have strongly confirmed the important role of EndMT in diabetic renal fibrosis, and many classical signaling pathways are crucial to this process. All three TGF-β isoforms (TGF-β1, TGF-β2, and TGF-β3) are confirmed to regulate EndMT in diabetic kidney disease (DKD), and the latest research has shown that TFPI2 contributes to diabetic renal fibrosis and EndMT by positively regulating the TGF-β/Smad signaling pathway [36]. The Wnt/β-catenin pathway is also activated in DKD, and the inhibition of C3a/C5a was found to suppress EndMT and renal fibrosis via the Wnt/β-catenin pathway in DKD [37]. Moreover, the deficiency of the endothelial glucocorticoid receptor was found to regulate EndMT via the Wnt/β-catenin pathway in DKD [38]. Of great clinical value, many drugs have been found to modulate EndMT by targeting specific pathways in DKD. Endothelial SIRT3 is a critical antifibrotic molecule that regulates EndMT via TGF-β/Smad pathway [39]. The SGLT-2 inhibitor empagliflozin was found to protect the kidney by maintaining SIRT3 levels and suppressing aberrant glycolysis [40]. Furthermore, the dipeptidyl peptidase-4 inhibitor linagliptin can inhibit TGF-β2-induced EndMT via microRNA 29a and act as an antifibrotic in DKD [41]. Similarly, AcSDKP also has an antifibrotic effect by inhibiting the EndMT process in DKD, and FGFR1 is essential in this process [42]. Nevertheless, the precise impact of EndMT on CaOx-induced kidney injury and renal fibrosis remains unclear. This study demonstrated that the inhibition of EZH2 suppresses EndMT, but the mechanism has not been explored in depth. Hence, it is valuable to further investigate the role of EZH2 in CaOx-induced kidney injury and renal fibrosis by regulating EndMT.

The mechanisms of ferroptosis include an imbalance of the antioxidant system, abnormal iron metabolism, and abnormal lipid metabolism. The SLC7A11/GSH/GPX4 axis is the main antioxidant system in ferroptosis, and the repression of the above axis can drive ferroptosis [43]. As ferroptosis-induced cell damage is reversible, targeting ferroptosis can be an effective treatment for various diseases. The protective impact of ferroptosis inhibition has been confirmed in kidney injuries. In cisplatin-induced AKI, the activation of VDR reduced the kidney injury by suppressing ferroptosis [44]. Additionally, quercetin was found to relieve I/R, or folic acid-induced kidney injury, by suppressing ferroptosis [45]. In our study, ferroptosis was found to activate in a kidney stone model. The use of the ferroptosis inhibitor Lip-1 suppressed the CaOx-induced ferroptosis and kidney injury.

Numerous investigations have concentrated on the epigenetic regulatory mechanisms involved in ferroptosis, and the epigenetic regulators in ferroptosis include histone modifications, lymphocyte-specific helicase, long non-coding RNA, deubiquitinase, and selenium [46]. The ubiquitination of H2B was confirmed to regulate ferroptosis by facilitating the transcription of SLC7A11 [47]. Moreover, the oncogene LINC00336 was found to inhibit ferroptosis by interacting with ELAVL1 in lung cancer [48]. However, it is unclear how EZH2 affects ferroptosis in kidney stones. In this study, RNA sequencing results showed 20 genes that were obviously upregulated in the shEZH2 group and were highly associated with ferroptosis. SLC7A11 was one of the genes screened out. Additionally, the CHIP assay confirmed that oxalate stimulation upregulated the EZH2/H3K27me3 levels at the SLC7A11 promoter. Furthermore, the knockdown of SLC7A11 rescued the shEZH2-induced upregulation of SLC7A11 levels and suppression of ferroptosis. These findings indicated that EZH2 regulates ferroptosis by directly downregulating the SLC7A11 expression level. Similar to the results of our experiments, EZH2 was reported to promote ferroptosis by suppressing SLC7A11 expression in a D-galactosamine-induced acute liver failure model [49]. Collectively, EZH2 inhibition may exert a protective effect on kidney stones by suppressing ferroptosis.

SOX4 is reported to regulate disease development through the activation of downstream gene transcription and epigenetic modification. For kidney injuries, SOX4 was found to upregulate in AKI and was abundantly enriched in the proximal tubules [50]. To investigate the causes of increased EZH2 in kidney stones, we used UCSC to predict the transcription factors that may regulate EZH2. SOX4 was one of the transcription factors screened out and was upregulated in kidney stones due to the RNA sequencing and western blot results. The SOX4/EZH2 axis-mediated H3K27me3 epigenetic silencing plays a crucial role in its regulatory effects [51]. However, the function of the SOX4/EZH2 axis in CaOx-induced kidney injury remains unclear. Correlation analysis revealed that SOX4 was positively correlated with EZH2. CHIP-qPCR analysis demonstrated that the SOX4/EZH2 axis directly regulated SLC7A11 in oxalate-stimulated HK-2 cells. Furthermore, C11 BODIPY staining and western blot results confirmed the impact of the SOX4/EZH2 axis on ferroptosis. Thus, the SOX4/EZH2 axis is a possible therapeutic target for CaOx crystal deposition-induced kidney injury.

## Conclusions

In conclusion, this study revealed the effect of EZH2 on CaOx-induced kidney injury and confirmed the protective effects of EZH2 inhibition on kidney injury and ferroptosis. Targeting the SOX4/EZH2/SLC7A11 pathway can be a novel therapeutic strategy to prevent CaOx crystal deposition-induced kidney injury.

### Supplementary Information

**Additional file 1: Table S1.** List of primers used for qRT-PCR. **Table S2.** The sequences of Chip primers. **Table S3.** Related to Fig. 6.

**Additional file 2: Figure S1. A** Immunofluorescence of CD31/αSMA and Ecadherin/αSMA. Green fluorescence represents CD31 or E-cadherin while red fluorescence represents αSMA. **B** MDA, GSH, and Fe2 + levels. **C** Immunofluorescence of SLC7A11, GPX4 and ACSL4 in vitro. **D** Western blotting analysis of SLC7A11, GPX4, ACSL4 and PTGS2. Scale bar = 50 µm. **P < 0.01 compared with the control group; ^#^P < 0.05 compared with the Gly group in **A**. *P < 0.05, **P < 0.01 compared with the control group; ^#^P < 0.05 compared with the Ox group in **B**–**D**.

**Additional file 3: Figure S2. A** Immunofluorescence of CD31/αSMA and Ecadherin/αSMA. **B** Dual-luciferase analysis. **C** Immunofluorescence of CD31/αSMA and Ecadherin/αSMA. Scale bar = 50 µm. **P < 0.01, ***P < 0.001 compared with the EZH2^fl/fl^ group; ^#^P < 0.05 compared with the Gly + EZH2^fl/fl^ group in **A**. ***P < 0.001 compared with the Over-NC group in **B**.

## Data Availability

The original contributions presented in the study are included in the article/ Supplementary Material. Further inquiries can be directed to the corresponding authors.

## Abbreviations

CaOx: Calcium oxalate
EZH2: Enhancer of zeste homolog 2
PRC2: Polycomb repressive complex 2
H3K27me3: Trimethylation of histone H3 lysine 27
SLC7A11: Solute carrier family 7, member 11
SLC3A2: Solute carrier family 3, member 2
GPX4: Glutathione peroxidase 4
ACSL4: Acyl-CoA synthetase long-chain family4
PTGS2: Prostaglandin-endoperoxide synthase 2
AKI: Acute kidney injury
CKD: Chronic kidney disease
TEC: Tubular epithelial cell
RCD: Regulated cell death
ROS: Reactive oxygen species
GSH: Glutathione
MDA: Malondialdehyde
BUN: Blood urea nitrogen
Cr: Creatinine
IHC: Immunohistochemistry
TEM: Transmission electron microscopy
CHIP: Chromatin immunoprecipitation
Gly: Glyoxylic
CD31: Platelet endothelial cell adhesion molecule-1
αSMA: Alpha smooth muscle Actin
E-cad: E-cadherin
