# Supplementary material for: The SOX4/EZH2/SLC7A11 signaling axis mediates ferroptosis in calcium oxalate crystal deposition-induced kidney injury
Source: J Transl Med. 2024 Jan 2;22:9. doi: 10.1186/s12967-023-04793-1 (PMC10763321; doi:10.1186/s12967-023-04793-1)
Supplement: Supplementary file 1 — Additional file 1: Table S1. List of primers used for qRT-PCR. Table S2. The sequences of Chip primers. Table S3. Related to Fig. 6. [file 12967_2023_4793_MOESM1_ESM.doc]

| Gene symbol | Forward | Reverse |
| --- | --- | --- |
| m-GPX4 | CATGCCCGATATGCTGAGTGTGG | TAGCACGGCAGGTCCTTCTCTATC |
| m-SLC7A11 | CCTCTGACGATGGTGATGCTCTTC | GGTGCTGAATGGGTCCGAGTAAAG |
| m-ACSL4 | ACTGGCGATATTGGAGAAT | CACATAGGACTGGTCACTT |
| m-EZH2 | AGCACAAGTCATCCCGTTAAAG | AATTCTGTTGTAAGGGCGACC |
| m-PTGS2 | TGTGACTGTACCCGGACTGG | TGCACATTGTAAGTAGGTGGAC |
| h-GPX4 | GGCTTCGTGTGCATCGTCACC | TTCACCACGCAGCCGTTCTTG |
| h-SLC7A11 | CGCAAGCACACTCCTCTACCAG | TCAGAGTGATGACGAAGCCAATCC |
| h-ACSL4 | TTGGGCATTCCTCCAAGTAG | CCTGCAGCCATAGGTAAAGC |
| h-EZH2 | AATCAGAGTACATGCGACTGAGA | GCTGTATCCTTCGCTGTTTCC |
| h-PTGS2 | TAAGTGCGATTGTACCCGGAC | TTTGTAGCCATAGTCAGCATTGT |

Table S1. List of primers used for qRT-PCR

Table S2. The sequences of Chip primers

| Target | Forward | Reverse |
| --- | --- | --- |
| SLC7A11 | GGAGCTTGTTGCTCAACTGAC | ACTCACAAAACAGTCGCATGTA |
| EZH2① | TGGCTAACACGGTGAAAC | CACCATTCTCCTGCCTCA |
| EZH2② | CCCCAACAGTTCATAGGT | GTGGCAATGCAGTTAAGAGTA |
| EZH2③ | CCTATCCTCCCCGCCTCC | GGTTCGCTGTAAGGGACGC |

Table S3, Transcription factors that may bind EZH2

| TFDP1 | KLF5 | BARX2 | HNF4G |
| --- | --- | --- | --- |
| ZBTB14 | KLF15 | ZFX | ZNF460 |
| EGR1 | KLF16 | ZNF384 | ZNF135 |
| LEF1 | ZNF148 | MEF2A | GLIS3 |
| SOX4 | MAZ | MEF2B | ZNF384 |
| SOX10 | SP9 | MEF2C | SNAI2 |
| E2F7 | SP3 | MEF2D |  |
| Wt1 | OVOL1 | HNF4A |  |

The TF list was assembled by predicting transcription factors that may bind to the EZH2 promoter region using UCSC (Minimum score=500)
