# Supplementary material for: The SOX4/EZH2/SLC7A11 signaling axis mediates ferroptosis in calcium oxalate crystal deposition-induced kidney injury
Source: J Transl Med. 2024 Jan 2;22:9. doi: 10.1186/s12967-023-04793-1 (PMC10763321; doi:10.1186/s12967-023-04793-1)
Supplement: Supplementary file 2 — Additional file 2: Figure S1. A Immunofluorescence of CD31/αSMA and Ecadherin/αSMA. Green fluorescence represents CD31 or E-cadherin while red fluorescence represents αSMA. B MDA, GSH, and Fe2 + levels. C Immunofluorescence of SLC7A11, GPX4 and ACSL4 in vitro. D Western blotting analysis of SLC7A11, GPX4, ACSL4 and PTGS2. Scale bar = 50 µm. **P < 0.01 compared with the control group; #P < 0.05 compared with the Gly group in A. *P < 0.05, **P < 0.01 compared with the control group; #P < 0.05 compared with the Ox group in B–D. [file 12967_2023_4793_MOESM2_ESM.doc]

**Additional file 2**


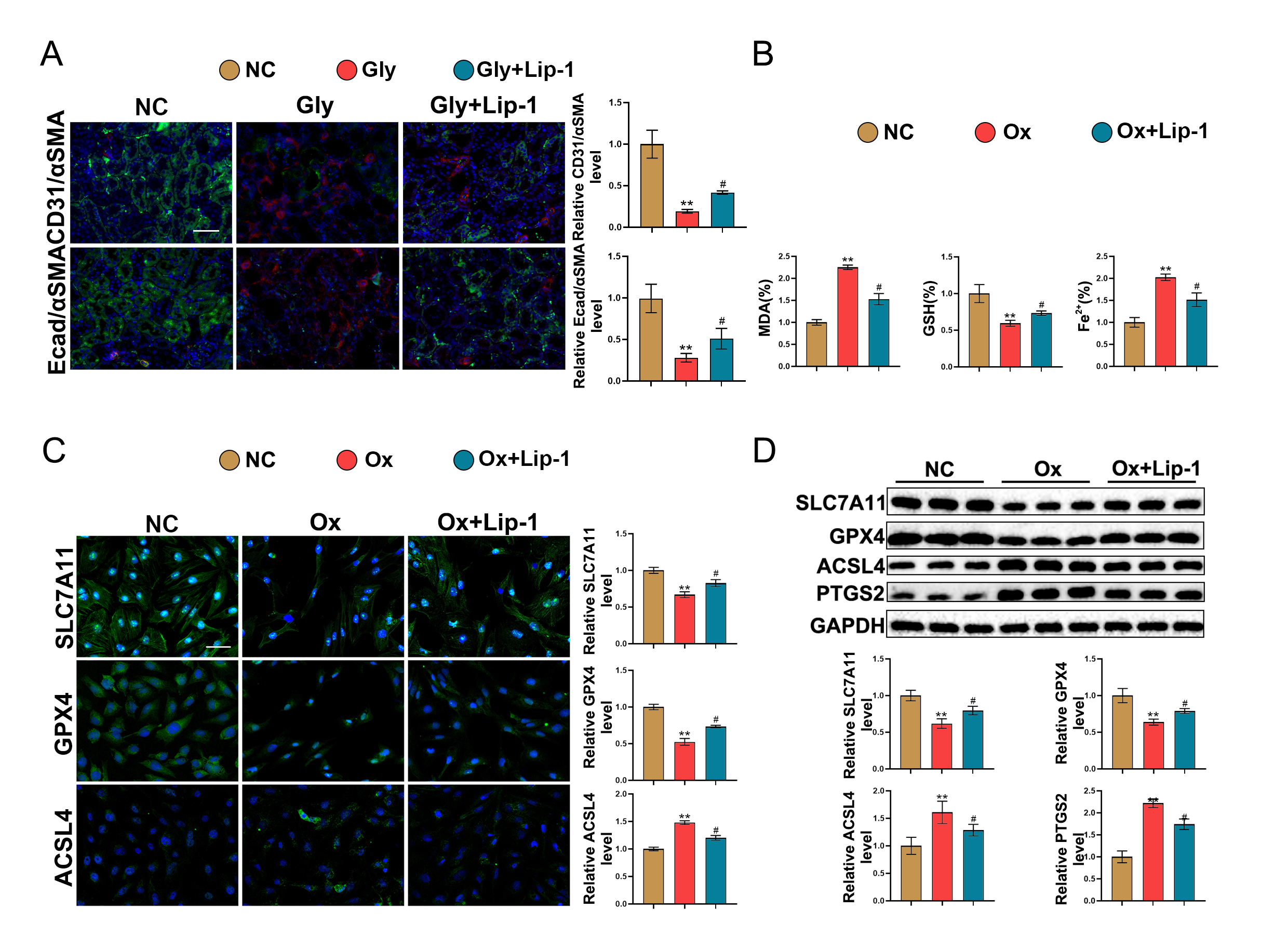


**Fig.S1. Lip-1 alleviates CaOx-induced EMT and EndMT *in vivo* and ferroptosis *in vitro*.**

A Immunofluorescence of CD31/αSMA and Ecadherin/αSMA. Green fluorescence represents CD31 or E-cadherin while red fluorescence represents αSMA. B MDA, GSH, and Fe2 + levels. C Immunofluorescence of SLC7A11, GPX4 and ACSL4 in vitro. D Western blotting analysis of SLC7A11, GPX4, ACSL4 and PTGS2. Scale bar = 50 µm. **P < 0.01 compared with the control group; ^#^P < 0.05 compared with the Gly group in A. *P < 0.05, **P < 0.01 compared with the control group; ^#^P < 0.05 compared with the Ox group in B–D.
