# Supplementary material for: The SOX4/EZH2/SLC7A11 signaling axis mediates ferroptosis in calcium oxalate crystal deposition-induced kidney injury
Source: J Transl Med. 2024 Jan 2;22:9. doi: 10.1186/s12967-023-04793-1 (PMC10763321; doi:10.1186/s12967-023-04793-1)
Supplement: Supplementary file 3 — Additional file 3: Figure S2. A Immunofluorescence of CD31/αSMA and Ecadherin/αSMA. B Dual-luciferase analysis. C Immunofluorescence of CD31/αSMA and Ecadherin/αSMA. Scale bar = 50 µm. **P < 0.01, ***P < 0.001 compared with the EZH2fl/fl group; #P < 0.05 compared with the Gly + EZH2fl/fl group in A. ***P < 0.001 compared with the Over-NC group in B. [file 12967_2023_4793_MOESM3_ESM.doc]

**Additional file 3**


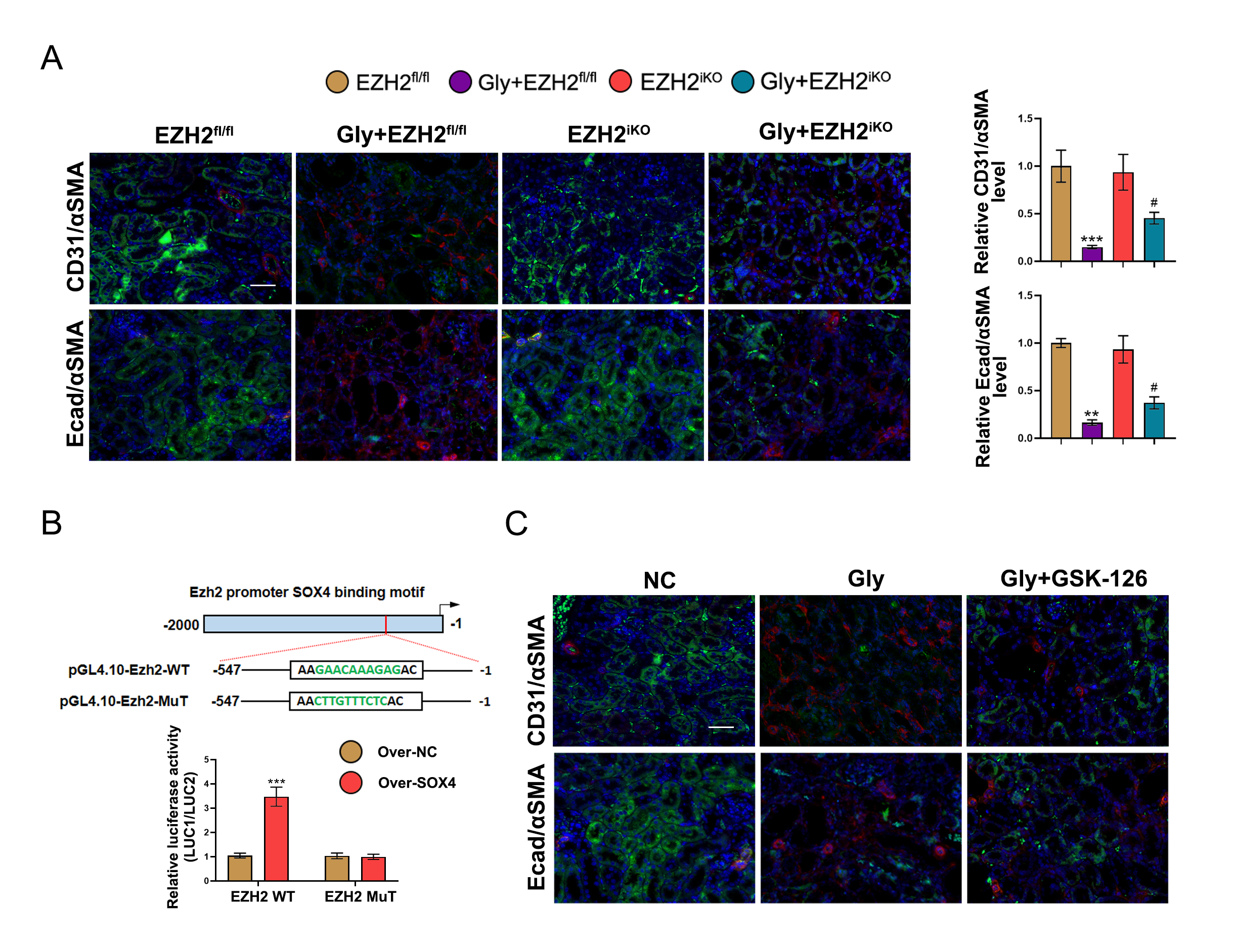


**Fig.S2. The effect of EZH2 knockout and EZH2 inhibitor GSK-126 on EMT and EndMT *in vivo*.**

A Immunofluorescence of CD31/αSMA and Ecadherin/αSMA. B Dual-luciferase analysis. C Immunofluorescence of CD31/αSMA and Ecadherin/αSMA. Scale bar = 50 µm. **P < 0.01, ***P < 0.001 compared with the EZH2fl/fl group; ^#^P < 0.05 compared with the Gly + EZH2^fl/fl^ group in A. ***P < 0.001 compared with the Over-NC group in B.
